# Epidemiology of Molar Furcation Defects: A Multi‐Center Study on Prevalence, Severity, and Risk Indicators

**DOI:** 10.1111/jre.70023

**Published:** 2025-07-30

**Authors:** Georgios S. Chatzopoulos, Larry F. Wolff

**Affiliations:** ^1^ Division of Periodontology, School of Dentistry University of Minnesota Minneapolis Minnesota USA; ^2^ Faculty of Dentistry, Health Sciences Aristotle University of Thessaloniki Thessaloniki Greece

**Keywords:** distribution, furcation, occurrence, periodontitis, risk factor

## Abstract

In a large cohort of periodontitis patients, molar furcation involvement was highly prevalent but mostly of lower severity. Tooth‐specific factors are primary drivers of risk, with maxillary molars and second molars having significantly higher odds of being affected.

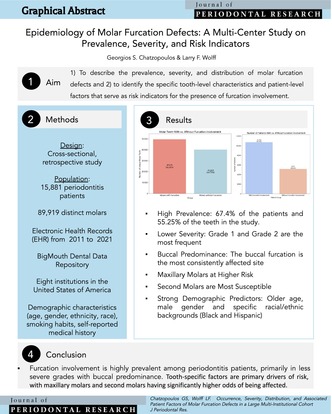

## 1 Introduction

Furcation involvement (FI) refers to bone resorption in the bifurcation and trifurcation of multirooted teeth [1]. Exposed furcation surfaces are highly susceptible to plaque accumulation, making effective plaque control and root debridement challenging [2]. Meta‐analyses consistently demonstrate that molars with FI have a significantly increased risk of tooth loss, particularly with more severe involvement [3, 4]. Previous studies have reported varied prevalences of FI, with some identifying age, smoking, and deep periodontal pockets as contributing factors [5, 6, 7]. Although general trends are understood, a large‐scale, multi‐institutional investigation using electronic health records (EHRs) provides a unique opportunity to gain deeper insights into FI prevalence and associated patient factors in a diverse clinical population. This study hypothesized that periodontal molar furcation defects vary in occurrence, severity, and distribution, and that these variances are related to patient‐specific parameters like age, systemic health, and smoking. Accordingly, this study aimed to: (1) describe the prevalence, severity, and distribution of molar furcation defects in a large, multi‐institutional cohort; and (2) identify the specific tooth‐level characteristics (arch, molar type) and patient‐level factors (age, gender, race) that serve as risk indicators for the presence of furcation involvement using a multilevel analytical approach.

## 2 Methods

This retrospective study analyzed EHRs from the BigMouth Dental Data Repository (2011–2021). Although standardized Current Dental Terminology (CDT) codes were used for reporting services across all participating institutions, the clinical data were documented by numerous providers (dental students, residents, and faculty), and no formal data harmonization procedures were applied to ensure diagnostic comparability across the sites. Clinical data on FI in all molar types were extracted. Only the initial assessment was used for patients with multiple examinations. Independent variables included demographic characteristics (age, ethnicity, race, gender, smoking, alcohol) and self‐reported medical conditions from patient questionnaires.

## 3 Results

A significant majority, 10 704 individuals (67.4%), exhibited FI in at least one tooth. Among affected patients (*n* = 10 351), less severe grades predominated: Grade 2 (44.05%) and Grade 1 (42.73%). Grade 3 and 4 were less common at 12.68% and 0.54%, respectively. At the tooth level, 55.25% of 89 919 M had FI, with Grade 1 being the most frequent (66.47%), followed by Grade 2 (29.25%). Grade 3 and 4 affected 4.16% and 0.11% of molars, respectively. The buccal furcation was consistently the most affected site in both maxillary and mandibular molars across all grades. Notably, the proportion of affected mandibular molars exhibiting lingual involvement increased as the furcation grade severity increased (Tables 1 and 2).

**TABLE 1:** Distribution of furcation involvement in maxillary molars: A breakdown by molar type, grade, and anatomic location.

Molar type	Highest overall grade	Total teeth	Buccal (count)	Buccal (%)	Mesial (count)	Mesial (%)	Distal (count)	Distal (%)
First molar	1	5.331	5.091	95.50	946	17.75	953	17.88
	2	2.343	2.209	94.28	968	41.31	991	42.30
	3	426	421	98.83	211	49.53	207	48.59
	4	12	11	91.67	5	41.67	5	41.67
Second molar	1	5.39	5.198	96.44	877	16.27	856	15.88
	2	2.474	2.349	94.95	987	39.89	1.064	43.01
	3	314	306	97.45	168	53.50	171	54.46
	4	10	9	90.00	4	40.00	6	60.00
Third molar	1	898	864	96.21	154	17.15	164	18.26
	2	450	436	96.89	158	35.11	161	35.78
	3	49	49	100.00	26	53.06	29	59.18
	4	1	1	100.00	1	100.00	0	0.00

**TABLE 2:** Distribution of furcation involvement in mandibular molars: A breakdown by molar type, grade, and anatomic location.

Molar type	Highest overall grade	Total teeth	Buccal (count)	Buccal	Lingual (count)	Lingual
First molar	1	5.369	5.293	98.58%	2.01	37.44%
	2	2.293	2.281	99.48%	1.465	63.89%
	3	339	338	99.71%	257	75.81%
	4	16	16	100.00%	11	68.75%
Second molar	1	5.131	5.071	98.83%	2.004	39.06%
	2	2.092	2.075	99.19%	1.306	62.43%
	3	274	274	100.00%	202	73.72%
	4	N/A	N/A	N/A	N/A	N/A
Third molar	1	1.114	1.099	98.65%	483	43.36%
	2	573	567	98.95%	405	70.68%
	3	52	52	100.00%	39	75.00%
	4	1	1	100.00%	1	100.00%

Fisher's exact test revealed significant associations between FI and older age groups (*p* < 0.0001), male gender (*p* < 0.0001), specific racial/ethnic groups, and numerous medical comorbidities (Appendix S2). Independent sample *t*‐tests showed that patients with FI were significantly older (mean 54.24 vs. 48.70 years, *p* < 0.0001) and had a higher mean number of missing teeth (2.34 vs. 2.03, *p* < 0.0001) and tooth loss during periodontal maintenance (0.52 vs. 0.36, *p* < 0.0001) compared to those without FI.

The results of the multilevel logistic regression analysis are presented in Appendix S3. At the tooth level, both arch and molar type were significant predictors of furcation involvement. Maxillary molars were found to have over twice the odds of furcation involvement compared to mandibular molars (OR = 2.15, 95% CI [1.90, 2.43]). Similarly, second molars had significantly higher odds of being affected compared to first molars (OR = 1.55, 95% CI [1.35, 1.78]). Although third molars showed a trend towards increased risk compared to first molars, this association did not reach statistical significance (*p* = 0.08). After accounting for these tooth‐level factors, several patient‐level characteristics remained significant independent risk indicators. The odds of furcation involvement increased substantially with age, with the 61–80 age group showing 2.90 times the odds compared to patients 18–40 years. Male gender was also associated with a higher likelihood of furcation defects (OR = 1.28). Furthermore, significant disparities were observed, with Black patients (OR = 1.48) and patients of Hispanic ethnicity (OR = 1.55) exhibiting significantly higher odds of furcation involvement compared to White and non‐Hispanic patients, respectively. In the final adjusted model, neither current smoking status nor diabetic status was found to be statistically significant predictors.

## 4 Discussion

This study represents one of the largest EHR analyses of FI, allowing for robust statistical assessment across diverse demographic, racial, ethnic, and medical history profiles. The anatomical distribution analysis revealed a consistent buccal predominance of FI in both maxillary and mandibular molars, aligning with known molar morphology [8]. The increasing prevalence of lingual involvement in advanced mandibular furcations suggests site‐specific progression, with important clinical implications. Our observed FI prevalence in periodontitis patients is higher than general population studies, which is expected given our focus on a higher‐risk patient cohort seeking treatment at university dental clinics [5, 7].

Older age and male gender emerged as significant independent predictors for FI, corroborating previous research on periodontal disease vulnerability [9]. Although local anatomical factors, such as a molar's position in the maxillary arch, are primary drivers of furcation risk, patient‐level characteristics like older age and male gender remain powerful, independent contributors. The persistence of significant disparities among racial and ethnic groups, coupled with the non‐significant findings for smoking and diabetes in the fully adjusted model, suggests a complex interplay where the effects of traditional risk factors may be mediated through strong demographic and tooth‐level variables. Significant independent associations between FI and specific racial/ethnic groups were also identified. These findings underscore potential disparities in furcation involvement, warranting further investigation into genetic predispositions, social determinants of health, and access to care. Race and ethnicity are well‐established predictors of health and quality of care, even after adjusting for socioeconomic status [10].

Records with missing data on the primary outcome of furcation involvement were excluded, potentially introducing selection bias. Findings in the current study have limitations related to its retrospective, cross‐sectional nature despite being strengthened by large numbers of patients and the multi‐institutional database. We therefore were not able to show a causal relationship between the examined factors and the FI.

## Author Contributions

Conception, acquisition of data, design: G.S.C. and L.F.W. Analysis, interpretation of results, drafting of the manuscript: G.S.C. Critical review of the manuscript: L.F.W. All authors approved the final version of the manuscript.

## Disclosure

Social media statement: Characteristics of furcation involvement in the population.

## Ethics Statement

This cross‐sectional, retrospective study received a determination from the University of Minnesota Institutional Review Board (STUDY00016576) that it did not constitute research involving human subjects.

## Consent

Informed consent was waived due to the retrospective design of the study.

## Conflicts of Interest

The authors declare no conflicts of interest.

## Supporting information


**Appendix S1:** jre70023‐sup‐0001‐AppendicesS1‐S3.docx.
**Appendix S2:** jre70023‐sup‐0001‐AppendicesS1‐S3.docx.
**Appendix S3:** jre70023‐sup‐0001‐AppendicesS1‐S3.docx.

## Data Availability

The data that support the findings of this study are available from the corresponding author upon reasonable request.
