# Supplementary material for: Epidemiology of Molar Furcation Defects: A Multi‐Center Study on Prevalence, Severity, and Risk Indicators
Source: J Periodontal Res. 2025 Jul 30;61(1):92–4. doi: 10.1111/jre.70023 (PMC12961364; doi:10.1111/jre.70023)
Supplement: Supplementary file 1 — Appendix S1: jre70023‐sup‐0001‐AppendicesS1‐S3.docx. Appendix S2: jre70023‐sup‐0001‐AppendicesS1‐S3.docx. Appendix S3: jre70023‐sup‐0001‐AppendicesS1‐S3.docx. [file JRE-61-92-s001.docx]

Appendix 1: Details of the study methodology

1. Description of the BigMouth Data Repository

The BigMouth Dental Data Repository is a multi-institutional, centralized database containing de-identified electronic health record (EHR) data from eight university-based dental clinics in the United States. The participating institutions include: Harvard University, University of Texas Health, University of California San Francisco, University of Colorado, Loma Linda University, University of Buffalo, The University of Iowa, and The University of Minnesota.

The repository was established to facilitate large-scale oral health research by aggregating real-world clinical data. The data for this study were extracted from records collected between 2011 and 2021. Clinical information, including diagnoses and procedures, was documented by a wide range of providers, including dental students, residents, and faculty members, as part of routine patient care.

For procedural and diagnostic coding, all participating institutions utilize standardized code sets, primarily the American Dental Association’s Current Dental Terminology (CDT) codes. This standardization allows for the aggregation of data based on specific treatments rendered. However, it is important to note that while the coding is standardized, the clinical application and documentation practices may vary between institutions and individual providers. For this study, no formal statistical data harmonization procedures were applied to reconcile potential site-to-site variations in clinical diagnostic patterns. This is acknowledged as a limitation inherent in using large-scale, real-world EHR data.

2. Definition of a periodontitis diagnosis

A patient was included if their record contained one or more of the specified Current Dental Terminology (CDT) codes indicating a history of non-surgical or surgical periodontal treatment.

3. Definitions of Covariates

All variables used in the final multilevel logistic regression model were selected a priori based on their established association with periodontal disease in the scientific literature.

Outcome Variable:

Furcation Involvement: The presence of a furcation defect in a molar tooth, assessed clinically. For the primary analysis, this was treated as a dichotomous variable (Present vs. Absent). A tooth was coded as "Present" if any grade of furcation involvement (Grade 1, 2, 3, or 4, according to Glickman, 1972) was recorded for any of its furcations.

Tooth-Level Predictor Variables:

Arch: The location of the molar, categorized as Maxillary (upper jaw) or Mandibular (lower jaw). Reference Group: Mandibular.

Molar Type: The specific type of molar, categorized as 1st Molar, 2nd Molar, or 3rd Molar. Reference Group: 1st Molar.

Patient-Level Confounder Variables:

Age: The patient's age at the time of the initial examination, categorized into three groups: 18-40 years, 41-60 years, and 61-80 years. Reference Group: 18-40 years.

Gender: The patient's self-reported gender, categorized as Male or Female. Reference Group: Female.

Race: The patient's self-reported race, categorized as White, Black, Asian, or Other. Reference Group: White.

Ethnicity: The patient's self-reported ethnicity, categorized as Hispanic or Non-Hispanic. Reference Group: Non-Hispanic.

Smoking Status: The patient's self-reported smoking status, categorized as Current Smoker or Never Smoker. Former smokers and those with unanswered status were excluded from this part of the analysis to ensure clarity. Reference Group: Never Smoker.

Diabetic Status: The patient's self-reported history of diabetes, categorized as Yes or No. Reference Group: No.

3. Explanation of the Analytical Approach

The unit of analysis for this study was the individual molar tooth. To account for the fact that multiple teeth are nested within each patient—and are therefore not independent observations—a multilevel logistic regression model was employed. This hierarchical modeling approach correctly handles the data structure by accounting for patient-level clustering.

The model was specified with the presence or absence of furcation involvement as the dichotomous outcome. The fixed effects included in the model were the tooth-level predictors (Arch, Molar Type) and the patient-level confounders (Age, Gender, Race, Ethnicity, Smoking Status, Diabetic Status). To account for the non-independence of observations, Patient ID was included as a random effect. This approach allows for the estimation of the effect of tooth-level characteristics while adjusting for patient-level factors and controlling for all unmeasured patient-specific variability.

All statistical analyses were performed using R software (version 4.2.1; R Foundation for Statistical Computing) with the package for mixed-effects modeling. A p-value of < 0.05 was considered statistically significant.

Reference: Glickman I. Clinical Periodontology: Prevention, Diagnosis, and Treatment of Periodontal Disease in the Practice of General Dentistry. 4th ed. Saunders; Philadelphia, PA, USA: 1972. pp. 242–245.

Appendix 2: Demographic, racial, ethnic, and medical history characteristics of the study population, stratified by presence of furcation involvement. The included demographic, racial, ethnic, and medical history variables were collected from self-reported patient questionnaires completed at the initial visit.

| **Variable** | **Category** | **Absence of Furcation Involvement** | **Presence of Furcation Involvement** | **P Value** |
| --- | --- | --- | --- | --- |
| **Age Group** | 18-40 | 1466 (42.44%) | 1988 (57.56%) | <0.0001 |
|  | 41-60 | 1807 (23.93%) | 5744 (76.07%) | <0.0001 |
|  | 61-80 | 1108 (20.68%) | 4251 (79.32%) | <0.0001 |
|  | >=81 | 85 (21.68%) | 307 (78.32%) | 0.0241 |
| **Gender** | Female | 2611 (28.75%) | 6472 (71.25%) | <0.0001 |
|  | Male | 1846 (24.20%) | 5782 (75.80%) | <0.0001 |
|  | UNANSWERED | 9 (20.00%) | 36 (80.00%) | 0.3988 |
| **Smoking Status (CIGARETTES)** | NO | 4021 (26.72%) | 11026 (73.28%) | 0.5637 |
|  | UNANSWERED | 106 (20.91%) | 401 (79.09%) | 0.0026 |
|  | YES | 339 (28.20%) | 863 (71.80%) | 0.2104 |
| **Race** | AMERICAN INDIAN ALASKAN NATIVE | 9 (39.13%) | 14 (60.87%) | 0.2351 |
|  | ASIAN | 206 (27.39%) | 546 (72.61%) | 0.6426 |
|  | BLACK | 800 (30.03%) | 1864 (69.97%) | <0.0001 |
|  | HISPANIC | 981 (26.59%) | 2709 (73.41%) | 0.9328 |
|  | Multiple Races | 455 (26.58%) | 1257 (73.42%) | 0.954 |
|  | OTHER RACE | 333 (29.26%) | 805 (70.74%) | 0.0405 |
|  | PACIFIC ISLANDER | 0 (0.00%) | 7 (100.00%) | 0.2009 |
|  | Undefined | 397 (25.95%) | 1133 (74.05%) | 0.5243 |
|  | WHITE | 1285 (24.52%) | 3955 (75.48%) | <0.0001 |
| **Ethnicity** | HISPANIC | 928 (29.56%) | 2211 (70.44%) | 0.0001 |
|  | Multiple Ethnicities | 164 (25.11%) | 489 (74.89%) | 0.391 |
|  | NON HISPANIC | 1846 (26.29%) | 5177 (73.71%) | 0.3665 |
|  | OTHERS | 266 (30.02%) | 620 (69.98%) | 0.0212 |
|  | Undefined | 1262 (24.97%) | 3793 (75.03%) | 0.0011 |
| **ANEMIA** | YES | 31 (37.80%) | 51 (62.20%) | 0.0324 |
| **ANEMIA** | NO | 4430 (26.62%) | 12214 (73.38%) |  |
| **BLEEDING DISORDERS** | YES | 1 (14.29%) | 6 (85.71%) | 0.6832 |
| **BLEEDING DISORDERS** | NO | 4460 (26.68%) | 12259 (73.32%) |  |
| **LYMPHOMA** | YES | 0 (0.00%) | 4 (100.00%) | 0.5793 |
| **LYMPHOMA** | NO | 4461 (26.68%) | 12261 (73.32%) |  |
| **HEMOTOLOGICAL DISORDERS** | YES | 94 (25.75%) | 271 (74.25%) | 0.7199 |
| **HEMOTOLOGICAL DISORDERS** | NO | 4343 (26.66%) | 11949 (73.34%) |  |
| **LEUKEMIA** | YES | 1 (16.67%) | 5 (83.33%) | 1.0 |
| **LEUKEMIA** | NO | 4460 (26.67%) | 12260 (73.33%) |  |
| **MULTIPLE MYELOMA** | YES | 0 (0.00%) | 3 (100.00%) | 0.5697 |
| **MULTIPLE MYELOMA** | NO | 4461 (26.68%) | 12262 (73.32%) |  |
| **MALIGNANT CONDITIONS** | YES | 150 (20.38%) | 586 (79.62%) | 0.0001 |
| **MALIGNANT CONDITIONS** | NO | 4294 (26.96%) | 11636 (73.04%) |  |
| **ANGINA** | YES | 54 (27.00%) | 146 (73.00%) | 0.9359 |
| **ANGINA** | NO | 4397 (26.66%) | 12096 (73.34%) |  |
| **CARDIOVASCULAR DISEASES** | YES | 287 (22.78%) | 973 (77.22%) | 0.0012 |
| **CARDIOVASCULAR DISEASES** | NO | 4174 (26.97%) | 11305 (73.03%) |  |
| **CONGENITAL HEART DISEASE** | YES | 12 (17.91%) | 55 (82.09%) | 0.127 |
| **CONGENITAL HEART DISEASE** | NO | 4443 (26.71%) | 12193 (73.29%) |  |
| **CORONARY HEART DISEASE** | YES | 104 (23.06%) | 347 (76.94%) | 0.084 |
| **CORONARY HEART DISEASE** | NO | 4352 (26.78%) | 11900 (73.22%) |  |
| **HISTORY OF ENDOCARDITIS** | YES | 13 (30.23%) | 30 (69.77%) | 0.6057 |
| **HISTORY OF ENDOCARDITIS** | NO | 4439 (26.67%) | 12208 (73.33%) |  |
| **HEART ATTACK** | YES | 70 (23.26%) | 231 (76.74%) | 0.1886 |
| **HEART ATTACK** | NO | 4374 (26.73%) | 11990 (73.27%) |  |
| **HIGH BLOOD PRESSURE** | YES | 956 (22.47%) | 3298 (77.53%) | <0.0001 |
| **HIGH BLOOD PRESSURE** | NO | 3498 (28.12%) | 8943 (71.88%) |  |
| **IMPLANT DEFIBRILLATOR** | YES | 91 (24.93%) | 274 (75.07%) | 0.4731 |
| **IMPLANT DEFIBRILLATOR** | NO | 4352 (26.69%) | 11954 (73.31%) |  |
| **RHEUMATIC FEVER** | YES | 12 (20.00%) | 48 (80.00%) | 0.3056 |
| **RHEUMATIC FEVER** | NO | 4441 (26.69%) | 12201 (73.31%) |  |
| **DIABETES** | YES | 337 (22.33%) | 1172 (77.67%) | 0.0001 |
| **DIABETES** | NO | 4105 (27.09%) | 11047 (72.91%) |  |
| **THYROID PROBLEMS** | YES | 235 (23.74%) | 755 (76.26%) | 0.0316 |
| **THYROID PROBLEMS** | NO | 4211 (26.86%) | 11465 (73.14%) |  |
| **GLAUCOMA** | YES | 398 (22.72%) | 1354 (77.28%) | 0.0001 |
| **GLAUCOMA** | NO | 4059 (27.15%) | 10894 (72.85%) |  |
| **AIDS** | YES | 0 (0.00%) | 3 (100.00%) | 0.5697 |
| **AIDS** | NO | 4463 (26.67%) | 12271 (73.33%) |  |
| **HIV** | YES | 18 (22.50%) | 62 (77.50%) | 0.4486 |
| **HIV** | NO | 4428 (26.67%) | 12177 (73.33%) |  |
| **SEXUALLY TRANSMITTED DISEASES** | YES | 55 (29.41%) | 132 (70.59%) | 0.4055 |
| **SEXUALLY TRANSMITTED DISEASES** | NO | 4392 (26.61%) | 12110 (73.39%) |  |
| **DIALYSIS** | YES | 33 (60.00%) | 22 (40.00%) | <0.0001 |
| **DIALYSIS** | NO | 4410 (26.54%) | 12209 (73.46%) |  |
| **KIDNEY** | YES | 230 (25.84%) | 660 (74.16%) | 0.5857 |
| **KIDNEY** | NO | 4228 (26.71%) | 11602 (73.29%) |  |
| **RENAL FAILURE OR INSUFFICIENCY** | YES | 96 (44.86%) | 118 (55.14%) | <0.0001 |
| **RENAL FAILURE OR INSUFFICIENCY** | NO | 4363 (26.44%) | 12138 (73.56%) |  |
| **ARTHRITIS** | YES | 693 (22.60%) | 2374 (77.40%) | <0.0001 |
| **ARTHRITIS** | NO | 3769 (27.62%) | 9879 (72.38%) |  |
| **LUPUS** | YES | 20 (27.40%) | 53 (72.60%) | 0.8947 |
| **LUPUS** | NO | 4427 (26.65%) | 12183 (73.35%) |  |
| **OSTEOPOROSIS** | YES | 202 (25.16%) | 601 (74.84%) | 0.3266 |
| **OSTEOPOROSIS** | NO | 4257 (26.78%) | 11638 (73.22%) |  |
| **DEMENTIA** | YES | 20 (35.71%) | 36 (64.29%) | 0.1312 |
| **DEMENTIA** | NO | 4437 (26.65%) | 12212 (73.35%) |  |
| **ANXIETY** | YES | 285 (28.00%) | 733 (72.00%) | 0.3237 |
| **ANXIETY** | NO | 4173 (26.59%) | 11520 (73.41%) |  |
| **DEPRESSION** | YES | 359 (28.05%) | 921 (71.95%) | 0.2499 |
| **DEPRESSION** | NO | 4099 (26.57%) | 11330 (73.43%) |  |
| **PARKINSON DISEASE** | YES | 6 (17.65%) | 28 (82.35%) | 0.2518 |
| **PARKINSON DISEASE** | NO | 4383 (26.94%) | 11887 (73.06%) |  |
| **MULTIPLE SCLEROSIS** | YES | 5 (23.81%) | 16 (76.19%) | 1.0 |
| **MULTIPLE SCLEROSIS** | NO | 4441 (26.66%) | 12216 (73.34%) |  |
| **SEIZURE OR EPILEPSY** | YES | 62 (39.24%) | 96 (60.76%) | 0.0005 |
| **SEIZURE OR EPILEPSY** | NO | 4379 (26.51%) | 12137 (73.49%) |  |
| **STROKE** | YES | 57 (26.76%) | 156 (73.24%) | 1.0 |
| **STROKE** | NO | 4391 (26.69%) | 12063 (73.31%) |  |
| **ORGAN TRANSPLANT** | YES | 28 (34.15%) | 54 (65.85%) | 0.1332 |
| **ORGAN TRANSPLANT** | NO | 4426 (26.63%) | 12193 (73.37%) |  |
| **ASTHMA** | YES | 90 (26.16%) | 254 (73.84%) | 0.9019 |
| **ASTHMA** | NO | 4302 (26.64%) | 11846 (73.36%) |  |
| **BRONCHITIS** | YES | 5 (15.15%) | 28 (84.85%) | 0.1681 |
| **BRONCHITIS** | NO | 4448 (26.65%) | 12241 (73.35%) |  |
| **CHRONIC BRONCHITIS OR EMPHYSEMA** | YES | 45 (22.84%) | 152 (77.16%) | 0.2562 |
| **CHRONIC BRONCHITIS OR EMPHYSEMA** | NO | 4361 (26.64%) | 12009 (73.36%) |  |
| **RESPIRATORY OR LUNG PROBLEM** | YES | 121 (24.35%) | 376 (75.65%) | 0.2572 |
| **RESPIRATORY OR LUNG PROBLEM** | NO | 4342 (26.73%) | 11904 (73.27%) |  |
| **SINUSITIS** | YES | 11 (28.95%) | 27 (71.05%) | 0.716 |
| **SINUSITIS** | NO | 4442 (26.62%) | 12242 (73.38%) |  |
| **SLEEP APNEA** | YES | 23 (22.55%) | 79 (77.45%) | 0.4313 |
| **SLEEP APNEA** | NO | 4418 (26.64%) | 12165 (73.36%) |  |
| **CHRONS DISEASE** | YES | 20 (26.67%) | 55 (73.33%) | 1.0 |
| **CHRONS DISEASE** | NO | 4422 (26.68%) | 12152 (73.32%) |  |
| **TUBERCULOSIS** | YES | 7 (41.18%) | 10 (58.82%) | 0.1764 |
| **TUBERCULOSIS** | NO | 4396 (26.57%) | 12151 (73.43%) |  |
| **CIRRHOSIS OR CHRONIC HEPATITIS** | YES | 2 (33.33%) | 4 (66.67%) | 0.6607 |
| **CIRRHOSIS OR CHRONIC HEPATITIS** | NO | 4439 (26.67%) | 12203 (73.33%) |  |
| **GASTRO INTESTINAL DISORDERS** | YES | 197 (24.97%) | 592 (75.03%) | 0.2837 |
| **GASTRO INTESTINAL DISORDERS** | NO | 4260 (26.73%) | 11679 (73.27%) |  |
| **HEPATITIS** | YES | 178 (24.96%) | 535 (75.04%) | 0.2992 |
| **HEPATITIS** | NO | 4284 (26.81%) | 11698 (73.19%) |  |

The single p-value represents the overall test of association from the chi-squared analysis

Appendix 3: Multilevel Logistic Regression Results for Tooth-Level and Patient-Level Risk Indicators of Molar Furcation Involvement

| Predictor Variable | Category | Odds Ratio (OR) | 95% Confidence Interval (CI) | P-value |
| --- | --- | --- | --- | --- |
| Tooth-Level Predictors |  |  |  |  |
| Arch | Maxillary (Ref: Mandibular) | 2.15 | 1.90 – 2.43 | <0.001 |
| Molar Type | 2nd Molar (Ref: 1st Molar) | 1.55 | 1.35 – 1.78 | <0.001 |
|  | 3rd Molar (Ref: 1st Molar) | 1.20 | 0.98 – 1.47 | 0.08 |
| Patient-Level Confounders |  |  |  |  |
| Age Group | 41-60 (Ref:18-40) | 1.75 | 1.50 – 2.04 | <0.001 |
|  | 61-80 (Ref: 18-40) | 2.90 | 2.55 – 3.30 | <0.001 |
| Gender | Male (Ref: Female) | 1.28 | 1.17 – 1.40 | <0.001 |
| Race | Black (Ref: White) | 1.48 | 1.30 – 1.68 | <0.001 |
|  | Asian (Ref: White) | 1.12 | 0.97 – 1.30 | 0.12 |
| Ethnicity | Hispanic (Ref: Non-Hispanic) | 1.55 | 1.38 – 1.74 | <0.001 |
| Smoking Status | Current Smoker (Ref: Never) | 1.19 | 0.99 – 1.43 | 0.06 |
| Diabetic Status | Yes (Ref: No) | 1.14 | 0.97 – 1.34 | 0.11 |

The model predicts the odds of furcation involvement for an individual molar and accounts for the clustering of teeth within patients. All predictor variables were selected a priori based on scientific evidence.
